# *Bacteroides fragilis* Enterotoxin Induces Autophagy through an AMPK and FoxO3-Pathway, Leading to the Inhibition of Apoptosis in Intestinal Epithelial Cells

**DOI:** 10.3390/toxins15090544

**Published:** 2023-09-03

**Authors:** Su Hyuk Ko, Jun Ho Choi, Jung Mogg Kim

**Affiliations:** 1 Barshop Institute for Longevity and Aging Studies, University of Texas Health Science Center, San Antonio, TX 78229, USA; kosh@uthscsa.edu; 2 Department of Cell Systems and Anatomy, University of Texas Health Science Center at San Antonio, San Antonio, TX 78229, USA; 3 Department of Microbiology, Hanyang University College of Medicine, Seoul 04763, Republic of Korea

**Keywords:** autophagy, *Bacteroides fragilis*, enterotoxin, intestinal epithelial cells

## Abstract

Macroautophagy/autophagy is essential for preserving cellular homeostasis by recycling nutrients and removing spoiled or aged proteins and organelles. It also has an essential role in defense mechanisms against microbial infections. However, the role of autophagy in enterotoxigenic *Bacteroides fragilis* infection remains largely unknown. In this study, we explored the role of *B. fragilis* enterotoxin (BFT) in the autophagic process of intestinal epithelial cells (IECs). The LC3-I of human HCT-116 IECs was converted to LC3-II by BFT stimulation. In addition, BFT-exposed cells showed the decreased expression of p62 in a time-dependent manner and increased levels of ATG5 and ATG12 gradually. Evidence of an enhanced autophagic process was supported by autophagosomes co-localized with LC3-lysosome-associated protein 2 in BFT-stimulated cells. The AMP-activated protein kinase (AMPK) and Forkhead box O3 (FoxO3a) axis were required for BFT-induced autophagy activation. In contrast with the activation of autophagy at 3–6 h after BFT exposure, IECs induced apoptosis-related signals at 12–48 h. HCT-116 IECs suppressing the formation of autophagosomes significantly activated apoptosis signals instead of autophagy early after BFT exposure. These data suggest that BFT can activate autophagy through the AMPK-FoxO3a pathway and the autophagy may suppress apoptosis during early exposure of IECs to BFT.

## 1. Introduction

Autophagy is an intracellular mechanism for regulating cellular homeostasis whereby lysosomes target and degrade dysfunctional proteins, lipids, and organelles [1]. The autophagic process initiates with the formation of a crescent-shaped double-membrane structure around a target substance and forms an autophagosome containing cytoplasmic cargo. Subsequently, autophagosomes and lysosomes fuse to form autolysosomes, and substances inside the cargo are degraded [2,3]. Autophagy-related genes (ATGs) are known to initiate vesicle formation. ATG8 is a ubiquitin-like (Ubl) protein that undergoes proteolytic processing before modifying the lipid phosphatidylethanolamine (PE). ATG12 is a second Ubl protein and is attached covalently to ATG5. Covalently linked ATG12-ATG5 proteins recruit ATG16 to generate phagophores forming double-membrane autophagosome engulfing intracellular cargo. Microtubule-associated protein 1A/1B-light chain 3 (LC3), the mammalian homolog of ATG8, can be converted to the microtubule-associated 1A/1B-light chain 3 protein-II (LC3-II) by conjugation with PE. LC3-II, a standard autophagosome marker, is present on inner and outer membranes, serving as a recognition site for LC3-binding chaperones such as p62/sequestosome-1 (SQSTM1) that deliver their cargo to autophagosomes [4]. The fusion of the autophagosomes with lysosomes forms autolysosomes, which contribute to lysosomal acid hydrolases. Then, lysis of the autophagosome inner membrane and breakdown of the cargos occurs in the autolysosome. The dynamic process, including all steps, is termed autophagic flux [1,3,4,5].

In addition to the role of autophagy in cellular homeostasis, recent studies revealed that autophagic machinery plays an important role in immune responses to several stimulators. For example, autophagy not only regulates inflammation but is also associated with degrading intracellular pathogens [6,7,8]. Pathogen-associated molecular patterns (PAMPs) and pathogen-induced damage-associated molecular patterns (DAMPs) are the primary mediators of bacteria-induced autophagy. Autophagy events, called xenophagy, are cell-autonomous innate immunity defense mechanisms that can selectively capture bacteria depending on PAMPs and DAMPs localization during infection [7,8]. Cell surface recognition of PAMPs and DAMPs promotes rapid and localized autophagic machinery recruitment. Inflammation-involved transcription factors such as transcription factor EB and nuclear factor kappa B (NF-κB) can stimulate autophagic gene expression and then regulate autophagic activity [9,10]. We previously revealed that *Bacteroides fragilis* enterotoxin (BFT) activates the accumulation of autophagosomes but does not fuse autophagosomes and lysosomes in human endothelial cells [4]. However, the autophagy of intestinal epithelial cells (IECs), which are the first contact sites of enterotoxin secreted from intestinal bacteria, is not understood.

Enterotoxigenic *B. fragilis* (ETBF) secreting BFT (20-kDa heat-labile zinc-dependent metalloprotease enterotoxin) is known to be associated with inflammatory diarrheal disease, inflammatory bowel disease, and colorectal cancer [11,12]. BFT affects the function of IECs by binding to the IEC receptor that is not E-cadherin [13], causing morphological changes, weakening the barrier function, and increasing inflammatory signals of IECs [11,12,14,15]. Our previous studies have demonstrated that BFT activates a signaling pathway involving p38 mitogen-activated protein kinase, IκB kinase, NF-κB, and heme oxygenase-1 (HO-1), leading to the inhibition of the apoptosis in IECs [16]. In addition, BFT activates a signaling cascade involving p38, Nrf2, and sulfiredoxin (Srx)-1, followed by the regulation of apoptosis in IECs [17]. Previous results implicate that IECs have a defense mechanism against ETBF infection. However, detailed host defense mechanisms in the context of ETBF infection and BFT exposure remain elusive. The present study investigated a signaling cascade regulating the autophagic response to bacterial enterotoxin. We found that a BFT-induced signaling pathway, including AMP-activated protein kinase (AMPK) and Forkhead box class O3a (FoxO3a), plays a crucial role in the initiation of BFT-induced autophagy in human IECs. Furthermore, enhanced autophagy is associated with the protection of IECs from apoptosis. From these findings, we were able to generate a hypothesis that AMPK-FoxO3-mediated autophagy could prevent apoptosis during ETBF infections.

## 2. Results

### 2.1. BFT Upregulates Autophagic Flux in IECs

**Figure 1:**
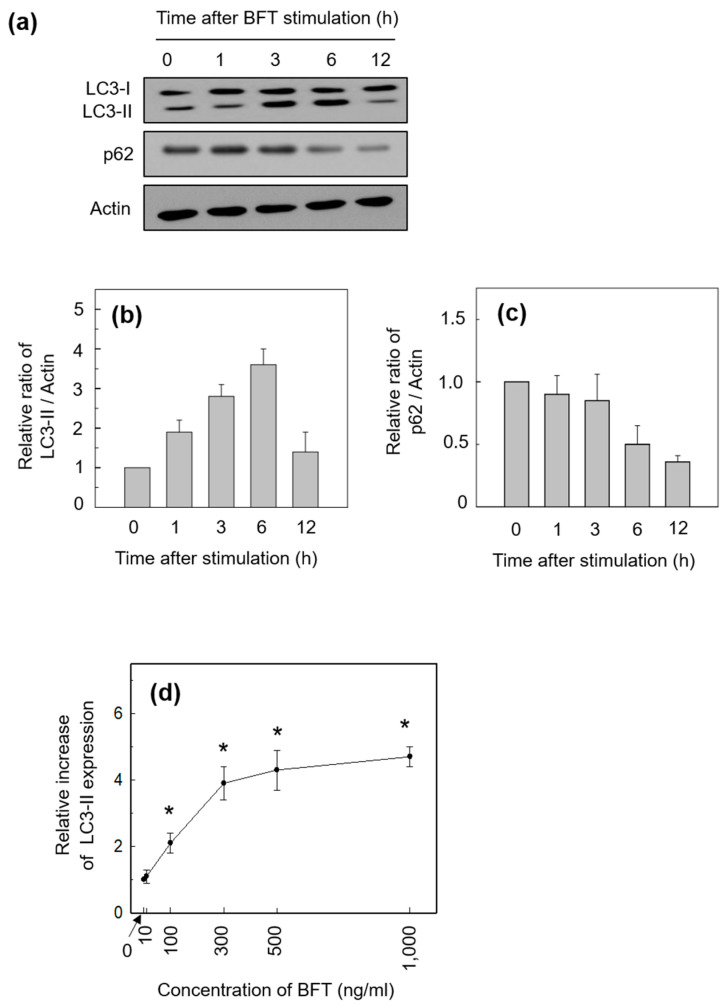
(**a**) HCT-116 cells were treated with BFT (300 ng/mL) for the indicated periods. Conversion of LC3-I to LC3-II, p62, and actin proteins were analyzed by immunoblot assays. Results are representative of three independent experiments. (**b**,**c**) Densitometric analysis of expressed LC3-II (**b**) and p62 (**c**) proteins on BFT-treated cells. Values represent the relative densities of each protein compared with actin (mean ± SD, *n* = 3). (**d**) HCT-116 cells were treated with the indicated concentrations of BFT for 6 h. The LC3-II expression was measured using an ELISA kit. Data were expressed as the mean increase relative to unstimulated controls ± SEM (*n* = 5). *, *p* value < 0.05 compared with the unstimulated control.

An early step in autophagy is the formation of double-membrane vesicles called phagophores, followed by elongation and engulfment of cytoplasmic cargo to form autophagosomes. Autophagosomes then fuse with lysosomes to form autolysosomes where cargo degradation occurs [2,3,5]. We first explored the autophagic process in BFT-exposed HCT-116 cells. In this experiment, the conversion of LC3-I to LC3-II, a standard autophagosome marker, was assessed using an immunoblot assay. HCT-116 cells showed enhanced conversion of LC3-I to LC3-II from 1 h post-BFT stimulation, indicating that the number of autophagosomes was increased in IECs (Figure 1a,b).

The p62 protein delivers target organelles to the autophagosome through binding with LC3, and fusion of the autophagosome with lysosome provides degradation of p62 and their cargo by lysosomal acid hydrolases. Therefore, the degradation of p62 signals is a marker of the fusion of autophagosomes with lysosomes. As shown in Figure 1a,c, we confirmed the gradual reduction in the protein levels of p62 after BFT stimulation in HCT-116 cells. Together with the increased formation of autophagosomes by BFT exposure, these results indicate that ETBF infection enhances the autophagic flux in IECs. The magnitude of LC3-II expression was dependent on the concentration of stimulated BFT (Figure 1d). Based on this experiment, we determined the stimulated concentration of BFT for use in the following experiments to be 300 ng/mL.

**Figure 2:**
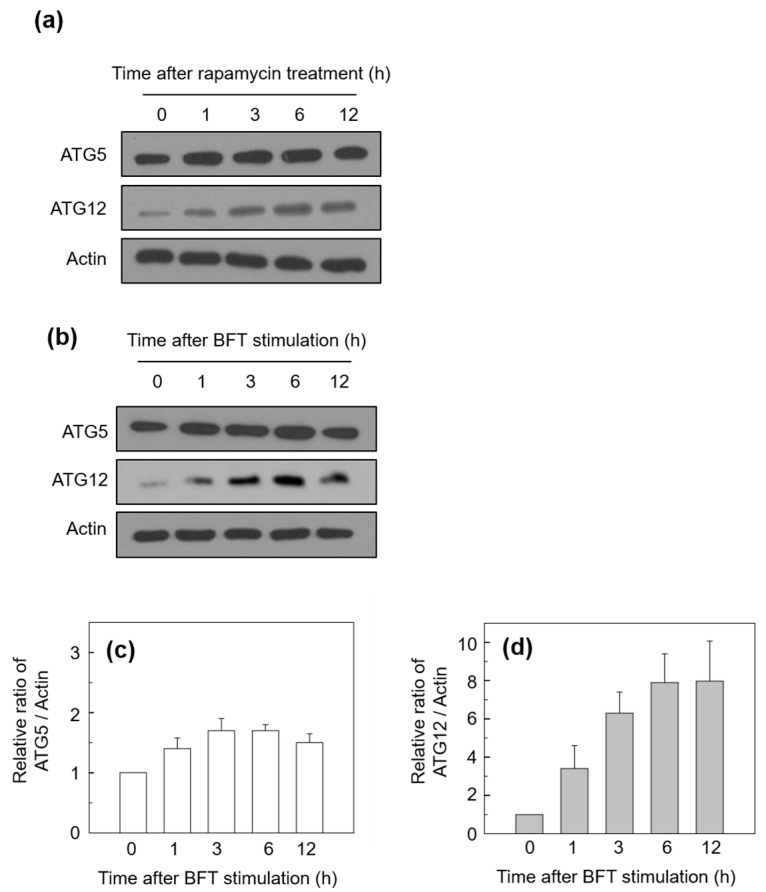
(**a**,**b**) HCT-116 cells were treated with BFT (300 ng/mL) for the indicated periods. The expression of each protein was analyzed by immunoblot assays. Results are representative of three independent experiments. (**c**,**d**) Densitometric analysis of expressed ATG5 (**c**) and ATG12 (**d**) proteins on BFT-treated cells. Values represent the relative densities of each protein compared with actin (mean ± SD, *n* = 3).

To investigate the initiation step of autophagy activated by BFT treatment, we measured the level of ATG5 and ATG12 proteins. Rapamycin, a well-known autophagy inducer, activates autophagy by suppressing the mammalian target of rapamycin complex 1 (mTORC1) [18]. We first compared the protein level of ATG5 and ATG12 in the treatment with rapamycin and that in BFT. As shown in Figure 2a,b, ATG5 and ATG12 protein expression in BFT-exposed cells was similar to the rapamycin-stimulated HCT-116 cells. The ATG5 proteins were slightly increased (Figure 2b,c). ATG12 proteins increased from 1 h after BFT stimulation and were gradually enhanced until 12 h post-stimulation (ATG12) in the BFT-treated cells (Figure 2b,d). These findings imply that exposure to BFT initiates the molecular mechanism of autophagy in HCT-116 cells.

**Figure 3:**
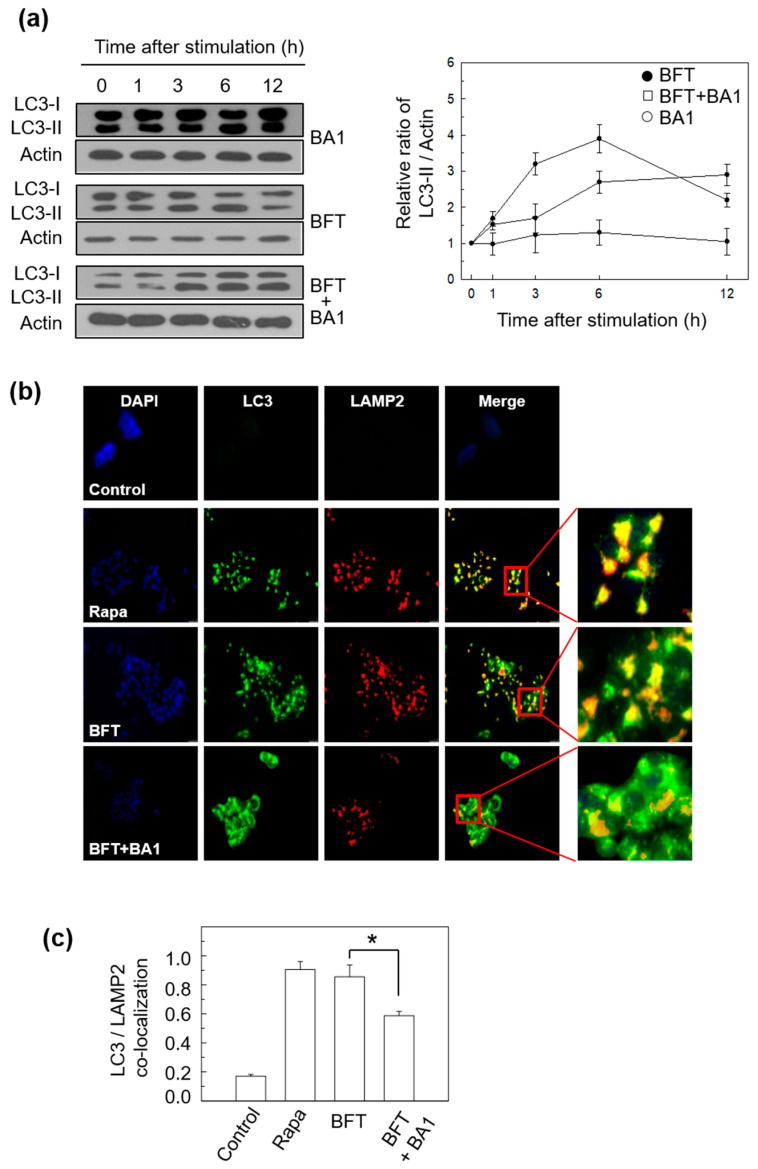

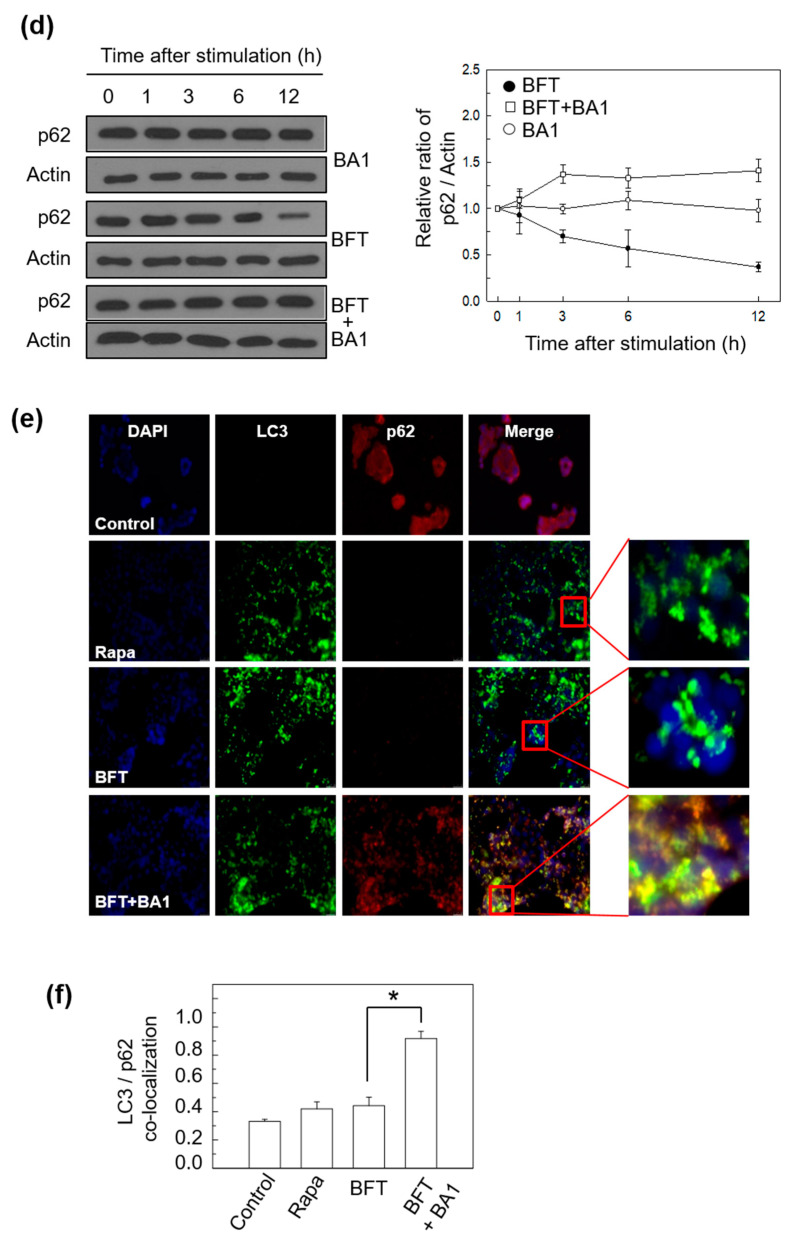
(**a**) HCT-116 cells were stimulated with BFT (300 ng/mL) and/or bafilomycin A1 (BA1, 20 nM) for the indicated period. The expression of each protein was analyzed by immunoblot assays. The densitometric analysis in the right panel is for the figures on the left (mean ± SD, *n* = 3). (**b**,**c**) HCT-116 cells were stimulated with BFT (300 ng/mL), rapamycin (Rapa, 200 nM), and BFT + BA1 (20 nM) for 6 h. Cells were treated with Abs against LAMP2 and LC3, and images were detected using fluorescence microscopy. Co-localization of LC3 and LAMP2 staining was quantified (mean ± SEM, *n* = 3). *, *p* value < 0.05. (**d**) Culture conditions were identical to Figure 3a. The expression of each protein was analyzed by immunoblot assays. The densitometric analysis in the right panel is for the figures on the left (mean ± SD, *n* = 3). (**e**,**f**) Culture conditions were identical to Figure 3b. Cells were treated with Abs against p62 and LC3, and images were detected using fluorescence microscopy. Co-localization of LC3 and p62 staining was quantified (mean ± SEM, *n* = 3). *, *p* value < 0.05. All images in Figure 3 are representative of three independent experiments.

We next determined whether chemical inhibition of autophagy could influence the BFT-mediated autophagic process. Bafilomycin A1 (BA1) is an inhibitor of the late phase of autophagy [19]. As shown in Figure 3a, the treatment of HCT-116 cells with BFT + BA1 (20 nM) combined showed a decrease in LC3-II expression compared to treatment with BFT alone. In this experiment, the expression level of LC3-II was increased with BFT + BA1 combined at 12 h of stimulation compared to the treatment of BFT alone. The reason for this seemed to be that, although BFT treatment increased the expression level of LC3-II, the fusion of autophagosomes with lysosomes did not occur due to BA1, and LC3 accumulated in the non-degraded autophagosomes. This observation was demonstrated in the previous report [20].

To confirm that the responses generated by HCT-116 cells against BFT stimulation were autophagic flux, other experiments were performed using immunofluorescence assay and image analysis. BA1 suppresses the maturation of autophagy cargos by blocking fusion between autophagosomes and lysosomes [19]. Consistent with the role of BA1 in suppressing autophagic maturation, treatment of HCT-116 cells with BA1 decreased co-localization between autophagosomes (labeled with LC3 Abs) and lysosome (labeled with Abs against LC3-lysosome-associated protein 2, LAMP2) under BFT-treated conditions (Figure 3b,c). In another experiment, the treatment of HCT-116 cells with BFT + BA1 combined did not decrease the protein expression of p62 compared with BFT alone (Figure 3d). In relation to this, BA1-induced accumulation of both LC3 and p62 was observed in BFT-exposed cells using an immunofluorescence assay (Figure 3e,f). These results indicate that BFT initiates the development of autophagosomes and the fusion of autophagosomes with lysosomes, indicating the complete autophagic process.

### 2.2. BFT-Induced Autophagic Flux Is Involved in FoxO3a Expression in IECs

**Figure 4:**
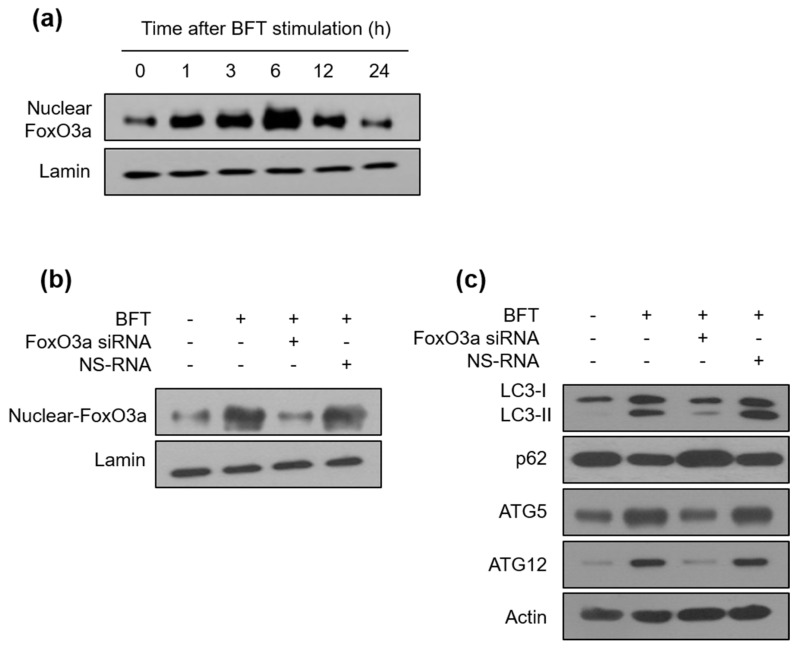
(**a**) HCT-116 cells were treated with BFT (300 ng/mL) for the indicated periods. FoxO3a and lamin B in the nuclear fraction were analyzed by immunoblotting (**left** panel). Densitometric analysis of expressed nuclear FoxO3a proteins on BFT-treated cells. (**b**,**c**) HCT-116 cells were transfected with FoxO3a-specific siRNA or nonsilencing siRNA (NS-RNA) as a control. After 48 h, BFT (300 ng/mL) was exposed to each group for 6 h. (**b**) The expression of nuclear-FoxO3a and lamin was analyzed by immunoblotting. (**c**) The expression of LC3, p62, ATG5, ATG12, and actin was analyzed by immunoblotting. All images are representative of three independent experiments.

**Figure 5:**
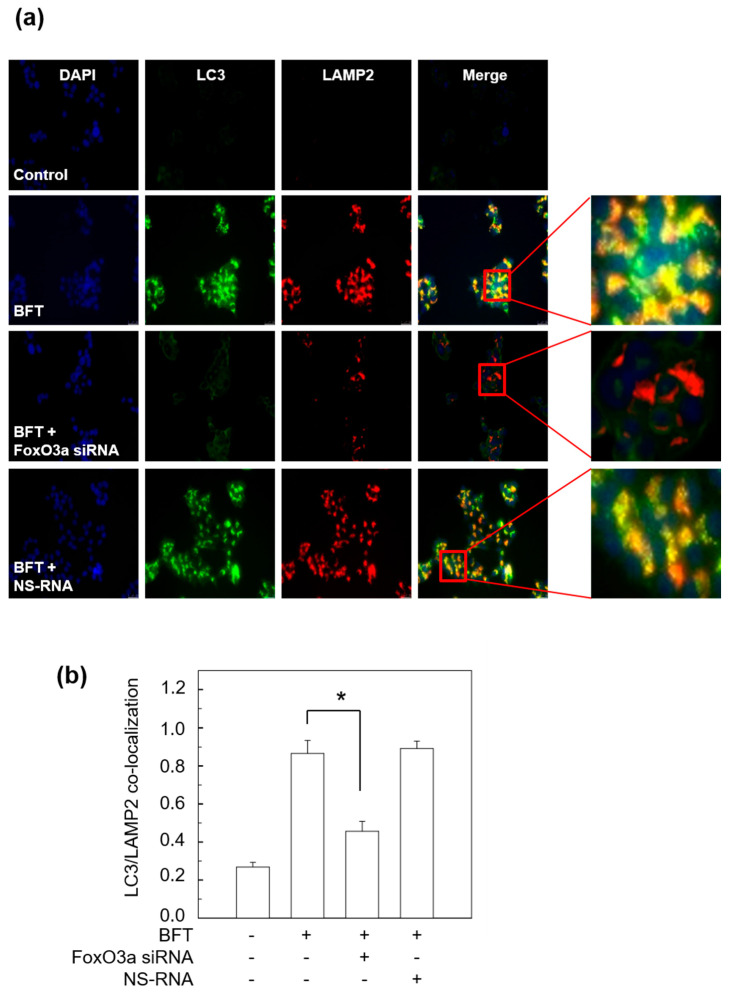
HCT-116 cells were transfected with FoxO3a-specific siRNA or nonsilencing siRNA (NS-RNA) as a control. After 48 h, BFT (300 ng/mL) was exposed to each group for 6 h. (**a**) HCT-116 cells were treated with Abs against LAMP2 and LC3 and analyzed by fluorescence microscopy. Results are representative of three independent experiments. (**b**) Co-localization of LC3 and LAMP2 staining was quantified (mean ± SEM, *n* = 3). *, *p* value < 0.05.

FoxO3a is known to control the expression of several genes associated with inflammation, oxidative stress resistance, apoptosis, and innate immune homeostasis [21]. The PI3K/Akt/FoxO3a axis regulates the expression of IL-10, an anti-inflammatory cytokine, in mycobacteria-infected macrophages [22]. To explore the role of FoxO3a in BFT-exposed IECs, we assessed FoxO3a protein levels in a nuclear fraction in BFT-exposed HCT-116 cells by immunoblot assay. As shown in Figure 4a, BFT augmented FoxO3a expression from 1 h post-stimulation, and the peak was noted at 6 h after stimulation, which gradually decreased. To analyze the association of FoxO3a expression with autophagic activation, we generated FoxO3a knockdown HCT-116 cells using FoxO3a siRNA, and a sufficiently downregulated expression level of FoxO3a was confirmed by immunoblot assay (Figure 4b). Interestingly, the increased level of the conversion rate of LC3-II from LC3-I by BFT exposure was decreased by FoxO3a siRNA transfection (Figure 4c). BFT failed the downregulation of the p62 expression level in the same situation (Figure 4c), indicating that FoxO3a has a crucial role in controlling autophagic flux by BFT stimulation. In this experimental model, BFT-induced increases in ATG5 and ATG12 expression were not observed in FoxO3a siRNA-transfected cells, indicating that FoxO3a may participate in the autophagic initiation regulatory mechanism by BFT (Figure 4c). Next, we visualized the co-localization rate between autophagosomes and lysosomes to double-check BFT-induced autophagic flux. Transfection with FoxO3a siRNA decreased the co-localization of LC3-LAMP2 compared with untransfected cells under BFT-treated circumstances (Figure 5a,b). These results suggest that FoxO3a-associated signaling regulates the autophagic flux in BFT-exposed IECs.

### 2.3. FoxO3a-Related Autophagy Is Connected with AMPK Activation in BFT-Exposed IECs

**Figure 6:**
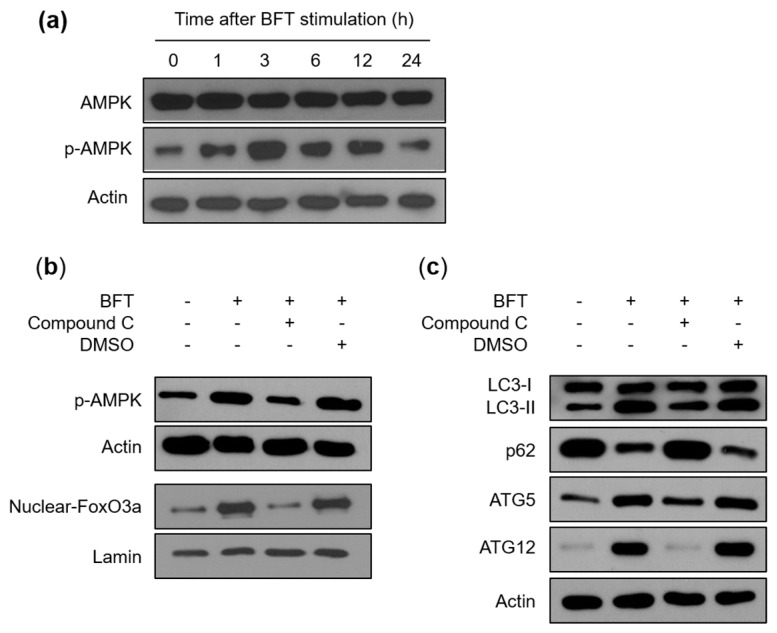
FoxO3a-medicated autophagy is associated with AMPK activation in BFT-exposed IECs. (**a**) HCT-116 cells were stimulated with BFT (300 ng/mL) for the indicated periods. AMPK, phospho-AMPK, and actin in the nuclear fraction were analyzed by immunoblotting. (**b**) HCT-116 cells were first incubated with compound C (10 μM) for 1 h and treated with BFT (300 ng/mL) for another 6 h. Expression of each protein was detected by immunoblot assays. (**c**) Experiments were identical in (**b**). The expression of LC3, p62, ATG5, ATG12, and actin was evaluated by immunoblotting. All images are representative of three independent experiments.

**Figure 7:**
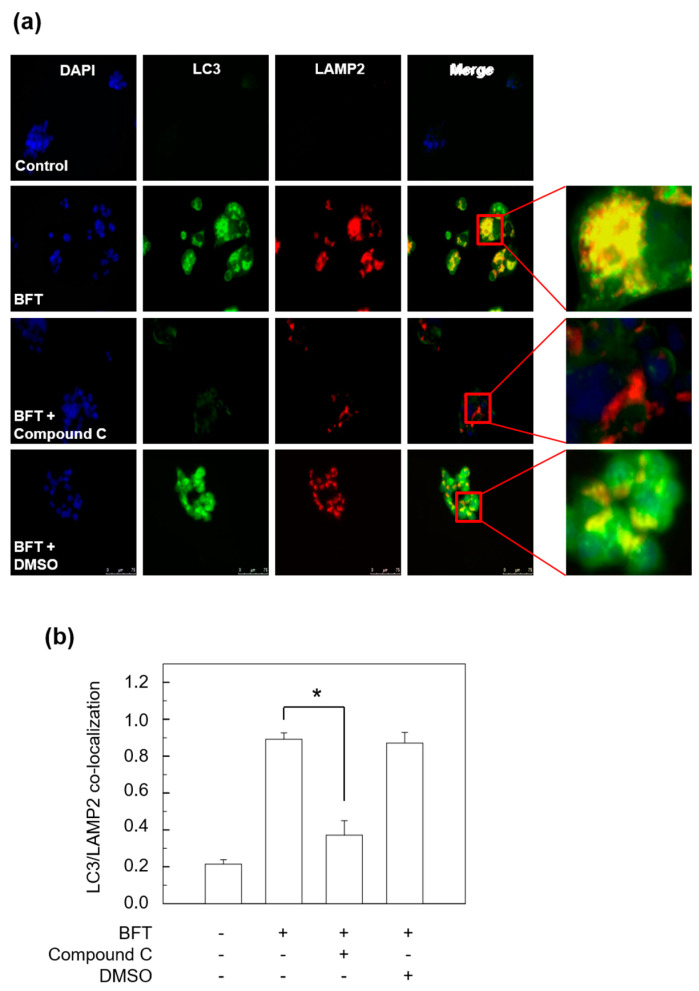
HCT-116 cells were first incubated with Compound C (10 μM) for 1 h and stimulated with BFT (300 ng/mL) for another 6 h. (**a**) HCT-116 cells were stained with Abs against LAMP2 and LC3 and then analyzed by fluorescence microscopy. Results are representative of three independent experiments. (**b**) Co-localization of LAMP2 and LC3 staining was quantified (mean ± SEM, *n* = 3). *, *p* value < 0.05.

The AMPK molecules are key regulators of cellular metabolism through modulating FoxO3a transcriptional activity [23]. We determined whether AMPK might be connected with BFT-induced FoxO3a-dependent autophagic flux. First, we investigated the expression of AMPK. An immunoblot assay revealed that the phosphorylated form of AMPK was enhanced from 1 h post-BFT treatment, whereas the total AMPK expression level was maintained (Figure 6a). Since AMPK activity is stimulated more than 100-fold by phosphorylation [24], our data implied that BFT can upregulate the AMPK metabolic activity in IECs. We next performed experiments on whether suppression of AMPK activity could change the autophagy in BFT-treated HCT-116 cells. Compound C, a well-known selective AMPK inhibitor [25], successfully suppressed the expression level of BFT-induced phosphorylated AMPK signals (Figure 6b). In this experimental model, the increased expression level of nuclear-FoxO3a by BFT-exposed cells was inhibited with compound C treatment, indicating that BFT-dependent nuclear FoxO3a requires AMPK activity (Figure 6b). Consistent with previous data, compound C, which can inhibit nuclear FoxO3a expression enhanced by BFT, sufficiently suppressed autophagic flux (LC3-II conversion and p62) and initiation (ATG5 and ATG12) in HCT-116 cells stimulated with BFT (Figure 6c). An immunofluorescence assay showed that enhanced co-localization between autophagosomes and lysosomes by BFT exposure was diminished by preincubation with compound C (Figure 7a,b). These results indicate that the activation of autophagy by IEC exposure to BFT requires the AMPK-FoxO3a axis.

### 2.4. Autophagy Delays BFT-Induced Apoptosis in IECs

**Figure 8:**
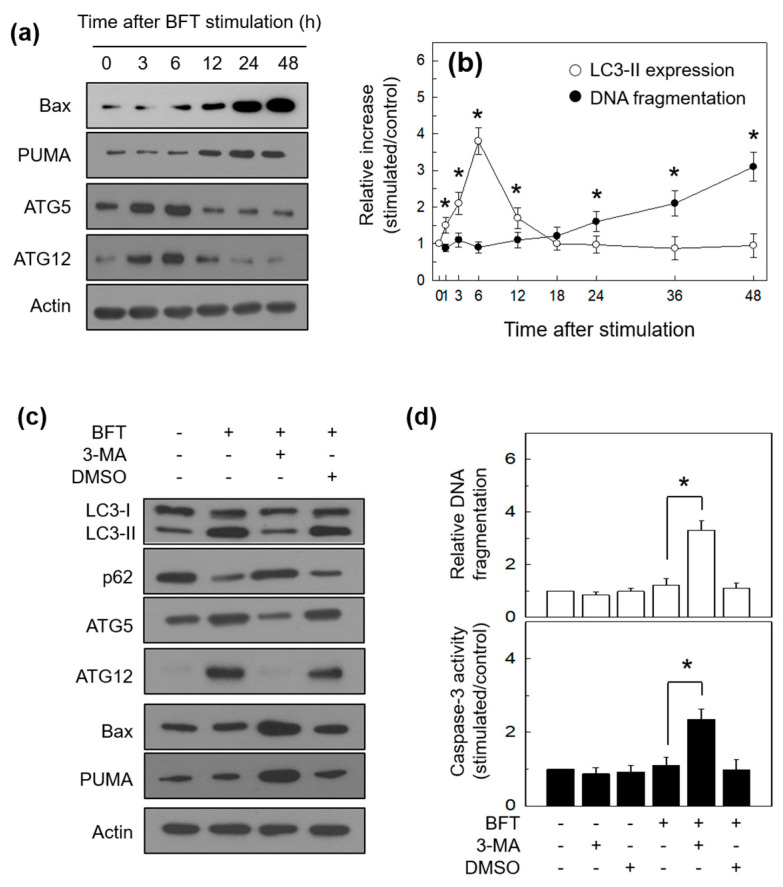
Association between autophagy and apoptosis in IECs stimulated with BFT. (**a**) HCT-116 cells were treated with BFT (300 ng/mL) for the indicated periods. Expression of autophagy proteins (ATG5 and ATG12) and apoptosis proteins (Bax and PUMA) were analyzed by immunoblot assays. (**b**) After adding BFT (300 ng/mL) to HCT-118 cells, DNA fragmentation and LC3-II expression were measured at the indicated periods of time using ELISA kits. Data are expressed as mean increase relative to unstimulated controls ± SEM (*n* = 3). *, *p* value < 0.05 (**c**) HCT-116 cells were preincubated with 3-methyladenine (3-MA, 10 mM) for 1 h and treated with BFT (300 ng/mL) for another 6 h. The protein expression of LC3, p62, ATG5, ATG12, Bax, PUMA, and actin was analyzed by immunoblotting. All images in (**a**,**c**) are representative of more than three independent experiments. (**d**) HCT-116 cells were preincubated with 3-MA for 1 h and then stimulated with BFT (300 ng/mL) for 12 h. DNA fragmentation and caspase-3 activity were measured by ELISA. Quantitative data analyses were expressed as mean increase relative to unstimulated controls ± SEM (*n* = 5). *, *p* value < 0.05.

Apoptosis and autophagy are known to be well-preserved programs that control cellular destiny [26]. Recent studies revealed that autophagy derived from nutrient deprivation or growth factor withdrawal could protect cells by preventing apoptosis [27,28]. To explore the association of apoptosis with autophagy under enterotoxin exposure, we measured the expression level of essential regulators of apoptosis such as Bax and PUMA. PUMA promotes apoptosis via activating the pro-apoptotic Bax protein and its interaction with anti-apoptotic Bcl-2 family members. This reaction then provokes caspase activation and DNA fragmentation characteristic of apoptosis [29]. In this study, HCT-116 cells showed the expression of both Bax and PUMA increased approximately 12 h post-BFT treatment, whereas ATG5 and ATG12 expression gradually decreased after a 6 h peak (Figure 8a). To confirm the time difference between autophagy and apoptosis, LC3-II expression and DNA fragmentation were evaluated using ELISA kits. The results showed a significant increase in LC3-II expression in the early period of BFT exposure with a maximum after 6 h. In contrast to autophagy, DNA fragmentation was first observed 24 h post-stimulation (Figure 8b). These results indicate that BFT can induce both autophagy and apoptosis processes by mechanisms that initiate sequentially.

Next, we inhibited autophagic flux using 3-methyladenine (3-MA), a selective autophagy inhibitor, and we then assessed the expression level of Bax and PUMA in BFT-exposed HCT-116 cells by immunoblot assay. LC3 conversion rate and expression peaks of ATG5 and ATG12 proteins, which were seen 6 h after BFT treatment, were suppressed by 3-MA treatment, and, simultaneously, p62 accumulation was observed (Figure 8c). Interestingly, pretreatment with 3-MA further increased the expression levels of Bax and PUMA after 6 h of BFT exposure (Figure 8c). In the next experiment, the pretreatment of HCT-116 cells with autophagy inhibitor 3-MA augmented the DNA fragmentation and caspase-3 activity 12 h post-exposure to BFT (Figure 8d).

**Figure 9:**
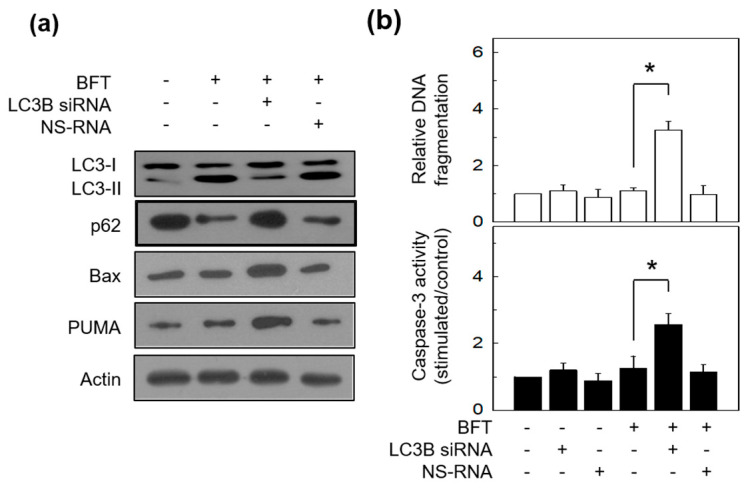
Relationships between autophagy and apoptosis in siRNA-transfected IECs under BFT exposure. (**a**) HCT-116 cells were transfected with LC3B-specific siRNA or nonsilencing siRNA (NS-RNA) as a control. After 48 h, BFT (300 ng/mL) was added to transfected and or untransfected cells for 6 h. The expression of LC3, p62, Bax, PUMA, and actin was analyzed by immunoblotting. All results are representative of three independent experiments. (**b**) Transfection conditions were identical to (**a**). BFT (300 ng/mL) was added to cells for 12 h. Measuring DNA fragmentation and caspase-3 activity were identical to (**a**). Data were expressed as mean increase relative to unstimulated controls ± SEM (*n* = 5). *, *p* value < 0.05.

These were double-confirmed in LC3-siRNA-transfected HCT-116 cells that stimulated BFT. LC3-siRNA-transfected HCT-116 cells showed further increased Bax and PUMA expression levels in response to BFT stimulation compared with NS-RNA transfection (Figure 9a). Consistent with this, LC3 siRNA augmented DNA fragmentation and caspase-3 activity 12 h post-exposure to BFT, in which definite apoptosis was not noted 12 h post-exposure in non-transfected cells under BFT-exposed circumstances (Figure 9b). These data imply that the onset of BFT-induced apoptosis may be shifted forward due to the absence of the autophagic process. Our data demonstrate that BFT can induce both autophagy and apoptosis and that autophagy can maintain IEC homeostasis by delaying apoptosis initiation.

## 3. Discussion

Autophagy, a process that preserves cellular homeostasis, acts as a specialized immunologic effector and regulates innate immunity to exert antimicrobial defense mechanisms [30]. Autophagosomes trap bacteria and ultimately induce lysosomal degradation [31]. Although several studies revealed that xenophagy, a selective autophagy that targets invading pathogens, reduces the survival of bacteria within the cell [32,33], mechanisms related to autophagy and bacterial enterotoxins are not well-known. The goal of the present study was to explore the role of autophagic flux and the host defense mechanisms related to it.

IECs constitute the first line of defense against enteric bacteria. This single-layer barrier is uniquely positioned to come in contact with bacteria and bacterial products such as BFT [34]. Our finding was that the enhanced autophagic flux in BFT-exposed IECs might indicate that intestinal epithelium can activate cellular defense mechanisms in response to bacteria-derived enterotoxins upon ETBF infection.

The present study showed the difference between IECs and endothelial cells in autophagy induced by BFT exposure. We demonstrated previously that BFT activates the accumulation of autophagosomes in human endothelial cells, and prevents the fusion of autophagosomes and lysosomes, indicating BFT-induced inhibition of complete autophagy in endothelial cells [4]. However, the present study revealed that BFT-exposed IECs not only induce the accumulation of autophagosomes but also enhance the fusion of autophagosomes and lysosomes, indicating BFT-induced complete autophagy in IECs. This difference in BFT-induced complete autophagy between IECs and endothelial cells may be derived from the types of cells that make contact with BFT.

Transcription factor FoxO is known to regulate the autophagy process [35]. Several studies have linked FoxOs to autophagy, and one of these suggests that FoxOs use epigenetic mechanisms to regulate autophagic activity [36]. For mammalian cells, FoxO3 induces the transcription of multiple autophagy genes, such as *LC3B* and *atg12*, by directly binding to their promoters [37]. Previously, we demonstrated that BFT exposure in IECs provokes specific signaling pathways that eventually result in the activation of transcription factors such as NF-κB and AP-1 [14,15,16]. Since early work revealed that lipopolysaccharide (LPS) and infection with *Citrobacter rodentium* target the FoxO3a transcription factor in intestinal epithelia [38], we hypothesized that BFT-induced autophagy requires FoxO3a in IECs. In the present study, the increased expression of FoxO3a by BFT treatment was observed in the nuclear fraction of HCT-116 cells. Furthermore, the BFT-induced LC3 conversion rate and expression of genes that contribute to the elongation of the isolated membrane were suppressed with FoxO3a siRNA transfection in HCT-116 cells. The decreased co-localization between autophagosomes and lysosomes in FoxO3a-siRNA-transfected cells under BFT exposure indicates that FoxO3a is an essential factor for regulating the autophagic response to ETBF infection.

Various stress conditions and their related pathways stimulate the induction of autophagy. The reduction in cellular energy activates AMPK by decreasing ATP/AMP ratio, then leads to phosphorylation and activation of the TSC1/2 complex, which induces autophagy through the inhibition of mTOR [39]. In the present study, we found that phosphorylated AMPK gradually increased by BFT treatment and abolished BFT-induced FoxO3a expression in nuclear fraction by artificial knockdown of p-AMPK. Moreover, decreased LC3 turnover rate, maintenance of p62 expression level, and low ATG5/12 in HCT-116 cells co-treated with BFT and compound C compared to the BFT alone group indicate that BFT requires AMPK activity for regulating autophagy. The results from the immunofluorescence images showing decreased LC3 signals and co-localization between autophagosomes and lysosomes by compound C treatment in BFT-exposed HCT-116 cells are consistent with the present data (Figure 6 and Figure 7). These results implicate that a signaling cascade involving AMPK and FoxO3a may play a crucial role in the initiation of BFT-induced autophagy in IECs. This study was conducted using only single cell line. Further studies are needed to prove the results demonstrated in animal experiments in vivo.

We previously demonstrated that apoptosis is delayed in BFT-exposed IECs [16,17,40]. Our finding resulted in the hypothesis that signals suppressing apoptosis are generated from BFT-exposed IECs. In a series of experiments to verify the hypothesis, we demonstrated that upregulated expression of cellular proteins, such as HO-1, Srx-1, and c-IAP-2, prevent apoptosis in BFT-exposed IECs [16,17,40]. Nevertheless, our data provoke a possibility that some other factors participate in cell protection when ETBF-producing enterotoxin contact IECs. For example, microbiota can both induce or inhibit apoptosis of IECs, which occurs spontaneously for maintaining barrier function [41]. In addition, complex crosstalk between autophagy and apoptosis in the epithelium could play a critical role in inflammatory diseases [42]. In this study, DNA fragmentation was not observed in BFT-exposed IECs 12 h before stimulation, when the autophagic process was fully manifested, but apoptosis was first observed at 24 h after stimulation and gradually increased up to 48 h after BFT stimulation. The 24 to 48 h time period of BFT post-stimulation corresponds to the later period of BFT stimulation, indicating that autophagy completely disappeared. In addition, a strategy for the inhibition of autophagic flux resulted in an augmentation of apoptosis in BFT-exposed IECs. In considering our results, we demonstrated that autophagy could delay apoptosis under BFT exposure in IECs. It is plausible that IECs can protect themselves by preventing cell death, such as apoptosis, through a raised autophagic process during ETBF infections. However, since this study focused on identifying the BFT-dependent pathway that can regulate autophagy in IECs, further research on the molecular mechanism between apoptosis and autophagy is required.

## 4. Conclusions

**Figure 10:**
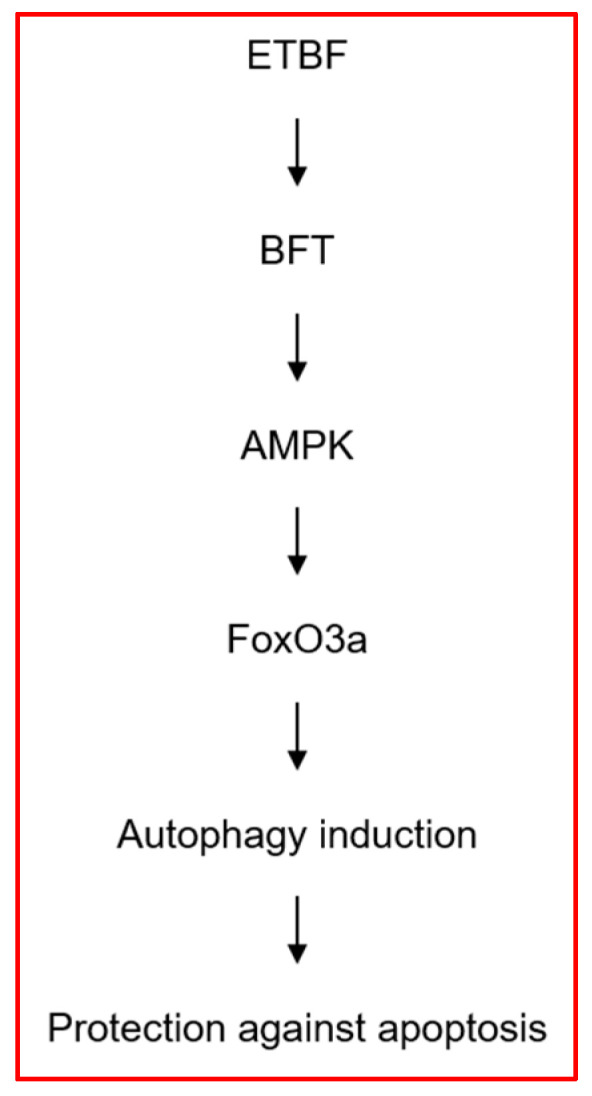
Schematic summary indicating how signal transduction by BFT induces autophagy leading to protection from apoptosis in intestinal epithelial cells.

We investigated a signaling cascade that regulates autophagic response to bacterial enterotoxin. In the present study, BFT increased the number of autophagosomes and accelerated the degradation of the p62 protein in IECs, ultimately increasing the autophagic flux. AMPK and FoxO3a signaling plays a crucial role in the initiation of BFT-induced autophagy in human IECs. In our experimental model, the inhibition of autophagy enhanced the apoptosis phenomenon at a relatively early stage in BFT stimulation (Figure 10). Our findings suggest that IECs activate an autophagy response to BFT exposure to suppress BFT-dependent apoptosis. These responses may contribute to cellular homeostasis by inhibiting apoptosis under infected conditions with enterotoxin-secreting ETBF.

## 5. Materials and Methods

### 5.1. Reagents

This study used reagents obtained from the commercial companies indicated below. Fetal bovine serum (FBS, Cat No. 12483-020) and TRIzol (GIBCO BRL, Gaithersburg, MD, USA); RPMI-1640 medium, Tween-20, bovine serum albumin (BSA), skim milk, and rapamycin (Sigma-Aldrich Chemical Co., St. Louis, MO, USA). Lipofectamine 2000 reagent (Invitrogen, Carlsbad, CA, USA); Abs against molecules such as LAMP-2 (sc-18822), lamin B (sc-374015), PUMA (sc-377015), and actin (sc-47778) (Santa Cruz Biotechnology, Santa Cruz, CA, USA); Abs against molecules such as ATG5 (Cat No. 9164), ATG12 (Cat No. 4180), FoxO3a (Cat No. 12829), AMPKα (Cat No. 5832), phospho-AMPKα (Cat No. 2535), and Bax (Cat No. 2772), and horseradish peroxidase-conjugated anti-rabbit IgG (Cat No. 7074) and anti-mouse IgG (Cat No. 7076) (Cell Signaling Technology, Inc., Beverly, MA, USA); anti-LC3 antibody (Cat No. NB100-2220, Novus Biologicals, Saint Charles, MO, USA); p62/SQSTM1 (Cat No. H00008878-M01, Abnova, Taipei City, Taiwan); DyLight 549- and Alexa Fluor 488-conjugated secondary Abs (Thermo Fisher Scientific, Waltham, MA, USA); Vectashield mounting medium with 4′,6-diamidino-2-phenylindole (DAPI, Vector Laboratories, Inc., Burlingame, CA, USA); compound C (HY-13418A, Medchemexpres, Monmouth, NJ, USA); bafilomycin A1 (Tocris Bioscience, Bristol, UK); 3-MA (Merckmillipore, Darmstadt, Germany)

### 5.2. Cell Culture and BFT Purification

Human HCT-116 intestinal epithelial cell lines (ATCC, CCL-247) was cultured in McCoy’s 5a medium with 10% FBS and antibiotics as described previously [16,17]. HCT-116 cells were incubated at 37 °C in a humidified atmosphere of 5% CO_2_. BFT was purified from culture supernatants of a toxigenic *B. fragilis* strain (ATCC 43858) as described previously [4,16,17].

### 5.3. Total Protein and Nuclear Protein Isolation

Total cellular proteins were obtained using an ice-cold protein extraction solution (iNtRON, Seongnam-si, Gyeonggi-do, South Korea) containing a 100 X phosphatase inhibitor (GenDEPOT, Baker, TX, USA). A commercial kit was used for the extraction of the nuclear protein fractions (NE-PER™ Nuclear and Cytoplasmic Extraction Reagents, Thermo Fisher Scientific, Cat No. 78833). Protein concentrations were measured by the Bradford method (Bio-Rad, Hercules, CA, USA) [4,16,17].

### 5.4. Western Blot Assays and ELISA

Western blots for identifying specific proteins in the present study were performed according to a previously established protocol [16,17]. Briefly, total cell lysates or nuclear proteins were separated by SDS-PAGE (Bio-Rad Laboratories Inc.) and electrotransferred onto a PVDF membrane (Millipore Corp., Billerica, MA, USA). The membranes were probed with specific primary Abs overnight at 4 °C and then incubated with secondary Abs. Specific protein bands were detected using the ECL system (GenDepot, Katy, TX, USA). To detect the protein bands, different gel concentration was used; 8% gel (nuclear-FoxO3a), 10% gel (p62, ATG5, ATG12, actin, lamin, AMPK, and phospho-AMPK), and 15% gel (LC3-I and -II, Bax, and PUMA).

An ELISA kit was used for determining the protein levels of LC3-II (LC3-II Quantitation, Cell Biolabs, Inc., San Diego, CA, USA). The assay was performed following the operation sheet provided by the manufacturer.

### 5.5. Experiments of Transfection

Lipofectamine 2000 reagent was used in transfections for HCT-116 cells and specific siRNA against the LC3B (sc-43390, Santa Cruz Biotechnology) and FoxO3a (Cell signaling Technology). A nonsilencing siRNA (NS-RNA, sc-37007, Santa Cruz Biotechnology) was used as a negative control. Specific siRNA-delivered cells were used for experiments 48 h after transfection. All experiments followed the protocols provided by the manufacturers [4,16,17].

### 5.6. Immunofluorescence and Image Analysis

Immunofluorescence and image analysis were performed according to a previously established protocol [4,16]. Briefly, HCT-116 cells were cultured on a chamber slide (Thermo Fisher Scientific) and fixed in methanol. Cells were incubated with the primary Abs (rabbit anti-LC3 and mouse anti-p62/SQSTM1 Abs or rabbit anti-LC3 and mouse anti-LAMP2 Abs). Cells were then incubated in fluorochrome-conjugated secondary Abs. Cells was treated to the DAPI, and images were detected using a DMI4000B fluorescence microscope (Leica Microsystems GmbH, Wetzlar, Germany) [4]. Analysis of co-localization was performed using JACoP (Just Another Co-localization Plugin) in the ImageJ program (NIH Image). A linear equation and Pearson’s coefficient were performed as previously described [43]. Results using this program ranged from +1 to –1, where 1 indicates a complete positive correlation and –1 is a negative correlation, and zero indicates no correlation.

### 5.7. Measurement of Apoptosis

Apoptosis was measured using a Cell Death Detection ELISA kit (Roche Diagnostics) for measuring DNA fragmentation and the Caspase-3 Colorimetric Assay kit (R&D Systems) for determining enzymatic activity of the caspase-3 in apoptotic cells, as described previously [16,17].

### 5.8. Statistical Analysis

Data in the present study are described as the mean ± standard deviation (SD) or mean ± standard error of means (SEM) and were evaluated by the Mann–Whitney *t*-test. A *p* value less than 0.05 was considered statistically significant.

## Data Availability

The data presented in this study are available in this article.
